# Progress of Gynæcology

**Published:** 1878-08

**Authors:** J. G. Westmoreland

**Affiliations:** Atlanta, Georgia


### PROGRESS OF GYNECOLOGY.

By J. G. WESTMORELAND, M.D., Atlanta, Georgia.

No branch of medical science has made more decided advancement in the past quarter of a century than that which relates to diseases peculiar to women. The harassing and often fatal difficulty, vesico-vaginal fistula, first engaged the attention of the American genius and philanthropist, Dr. J. Marion Sims, some thirty years ago; and others profiting by his labors, have afforded relief to many of this unfortunate class of females. From time to time improvements have been made in the manner of operating, and now vesical openings into the vagina are permanently cured by the ordinary gynecologist with as much certainty and ease as any disease of these organs.

The success of his operations gave Dr. Sims prominence as a specialist in the treatment of female disorders generally, requiring a larger field than his home in Alabama would afford. Even New York has not proved sufficient for his growing fame and ambition, and now he is being consulted by suffering females in England, France and Germany. His fame is well merited, he having worked a real and lasting benefit to humanity without humbuggery or deception. For this he will always be held in high esteem.

The success of Dr. Sims has awakened thought and investigation of other hitherto incurable female affections. The profession had for a long time been annoyed with the treatment of various troublesome symptoms, whose origin was not more perfectly understood than a century before. Indeed, the ancients seemed to have a more correct idea of the cause of some of these, than practitioners of the present age. For instance, the name given to certain symptoms by those practicing the healing art centuries ago, was said to be a misnomer thirty years since, but is now considered indicative of their true cause. Hysteria, the name by which a certain train of nervous symptoms are known, was given by the ancients, and certainly indicates disease of the uterus. Less than half a century ago this origin was disputed, but now all intelligent physicians know that uterine derangement leads to such nervous symptoms.

Advancement in gynecology has not only led to an acknowledgement of the origin of hysterical symptoms, but to the investigation and proper treatment of organic uterine disease upon which they depend. Not only so, but other symptoms of womb trouble, such as leucorrhœa and prolapsus are now, thanks to this advancement of science, rarely mentioned by intelligent practitioners as diseases to be treated. Even retroflexion, anteflexion, dysmenorrhœa, etc., are now being alluded to, and properly, we think, as the result of organic lesion.

Until comparatively a recent date, authors of standard works (who, by-the-by, fear the effect of innovations upon their sales) wrote knowingly of treating leucorrhœa by constitutional means. Even now dysmenorrhœa, from whatever cause, is treated by some as in the days of Dewees, with the so-called emmenagogues, without investigation as to the pathological condition of the uterus.

Pessaries, which sometimes, though rarely, serve as auxiliaries in the cure of uterine disease, are now looked upon as of doubtful efficacy, whereas they were once used as the only means of relief in the various forms of displacement.

Although abnormal positions of the organ may be the result of mechanical pressure, such as distended bladder, ovarian or other pelvic tumor, yet in a very large majority of such cases they depend upon organic lesion. Engorgement, interstitial deposits, etc., giving increase of specific gravity, tend very much to displacement; and adhesions, from chronic inflammation dependent upon the conjestion, are likely to render flexions more or less difficult of adjustment. Indeed, irregular deposit from inflammatory condition of the uterus may cause flexion which cannot be remedied without absorption of the semi-organized lymph, deposited, it may be, in one side of the cervix or body.

Leucorrhœal discharge, which may sometimes depend upon urethral or vaginal irritation, generally will be found to proceed from the uterine cavity, and will, then, cease only after intrauterine irritation has been allayed.

Various modes of rational treatment of the womb have been advised, to suit the various lesions to which the organ is subject. Congestion, which is often the precursor and common attendant of chronic metritis and endometritis, is relieved by local depletion. To this end, leeches, punctures or incisions, for the abstraction of blood, may be used successfully. Detergents, such as glycerine, are said to be also effectual for this purpose, when applied to the os and cervix by saturated cotton or wool. Advanced cases of chronic inflammation of the substance of the womb or its lining membrane, are relieved more promptly and perfectly by preceding more direct treatment with some of these depletory measures.

The important fact first to be remembered in connection with the permanent cure of endometritis, upon which displacements, leuchorrhœa and reflex hysterical symptoms may depend, is, that *like all other chronic inflammations there will be a tendency to return for a longer or shorter time after being relieved, according to the length of time which it has existed.* The failure to recognize and act upon this fact, leads to many of the discouraging unsuccessful attempts at cure by proper means. Patient and physician lose confidence in rational treatment when they find symptoms of disease return after being absent for months. Without recognition of this inevitable circumstance, there is no permanent benefit in any treatment. It is therefore proper to mention it in advance of any mode.

Scanzoni, the great pioneer and fearless innovator in the study of organic uterine disease, failed to give this point due consideration, and became discouraged in his effort at permanent relief. Under the name of "uterine catarrh," he gave to endometritis its proper importance in the production of its various symptoms and results. That pathognomonic limpid albuminoid flow from the os he clearly described, and showed his idea of its constancy in the disease by making it a foundation for the name. Had this pathologist and independent thinker observed more closely the plan of guarding against return, he would have been more hopeful of permanent cure.

A case when apparently cured must be watched for months, and when of long standing, years, in order to arrest any tendency to return before too firmly fixed again. Like other mucous membranes, this, when in a state of chronic inflammation, requires the direct application of remedies in order to a cure. All the plans of treatment on this line amount to about the same when properly carried out. The mode of application is about the only difference. Indeed, various articles are applied by the same practitioner at different times and under different circumstances, but all belong to the same general class of medicines—local alteratives, cathartics, or mild cauterants. Sulph. zinc, iodine, carbolic acid, nitrate of silver, etc., are the remedies, and the application is made in various ways, according to the peculiar views of practitioners. All recognize the importance of having the remedy applied to the whole intrauterine mucous membrane. In effecting this, there are two important difficulties to be met. These are uterine colic, when liquid preparations are suddenly thrown into the cavity, and the increase of irritation by any substance with which the medicine is incorporated for ready introduction, on account of remaining too long in contact with the inflamed mucous membrane. Hence, the syringe has been discarded by some on account of seeing their patients thrown into violent suffering from it; and those introducing a pledget of lint saturated with a medicated liquid, finding irritation increased by its presence in the cavity for a day or two, have sought a less ojectionable plan. The probe wrapped with medicated lint, introduced and promptly withdrawn, was found less objectionable. This seems to have been the plan most generally adopted by those not accustomed to use the syringe safely, till the medicated cloth tent was invented and brought into notice by Dr. Taliaferro. The tent is of course subject to the same objection that was found to the lint, if suffered to remain in the cavity too long. The difficulty may be avoided, however, by removing it early, say in an hour after its introduction.

The syringe, perhaps preferable to all other modes of making application, is nevertheless subject to objection on two accounts, both of which, however, may be effectually overcome by experience and care in its use. Not only may colic be induced occasionally, as above stated, by suddenly injecting any liquid into the uterine cavity, but when too much of an irritating remedy is thrown in, so as to produce sudden violent contraction called colic, it may be forced through the fallopian tubes, find its way to the peritoneum, and produce fatal efiects. Accounts of such result have been reported, but I have the evidence of more than a thousand instances that both difficulties may be avoided by injecting very slowly not more than fifteen or twenty drops of the liquid. This is a sufficient amount to give contact with the whole surface, and not enough, in case of colic, for any to find its way to the peritoneum.

With any judicious mode of application, promptly kept up, the chronic endometritis will gradually subside. It is generally, of several years standing before treatment is sought, and when the catarrh has ceased and all other evidences of disease have disappeared, the case is by no means permanently cured. In a few weeks, or it may be months, the catarrhal and hysterical or other nervous manifestations admonish the patient of returning disease, which must be met promptly in order to permanent relief. Owing to this tendency, patient and physician lose confidence in the treatment, and fail to give the case proper attention.

In the midst of this general discouragement in looking for a permanent cure, new plans of treatment have been sought by some, while others relapse into the old routine of tonics, alteratives and nervous stimulants, and content themselves in giving ease and comfort for the time, rather than pursue a laborious plan of treatment with what seems a doubtful prospect of permanent relief, or adopt more heroic means with even less probability of success. In this state of affairs Dr. Battey conceived the idea that uterine disease ceased with the appearance of the menopause, and hence concluded to cure permanetly the trouble by "normal ovariotomy." This operation consists in removing healthy ovaries, in order to cure uterine disease, by bringing on the change of life artificially. This dangerous measure was promulgated as a last resort, to meet the cases which had been treated by direct local means ineffectually, many of which were to be found, on account of the discouraging tendency to return above alluded to.

That menstruation is generally arrested, or that disease is eradicated from the uterus by the removal of healthy ovaries, recorded facts fail to prove. Of one thing, however, we are certain: disease of the uterus is not always relieved by the natural menopause. The writer has treated several cases much beyond this period of life; and one is distinctly remembered, which received treatment before the change of life and some ten years afterward also, for chronic uterine inflammation. The theory, therefore, that normal ovariotomy will produce the menopause or change of life, and that the change of life will work a cure of uterine irritation, chronic inflammation or ulceration, we think has not been sustained by facts.

The question then arises, "is there a field for normal ovriotomy?" We think there is a small field, notwithstanding the fatality of the procedure. Malformations, in which the ovaries exist without any womb, and cases where the ovaries are present with irremedial occlusion of the vagina require spaying. These are cases in which the risk of life by the operation is overbalanced by the tortures of an existence which will probably be terminated by the difficulty long before the ovaries lose their functions. Such cases we have seen, and in them advised

"normal ovariotomy," but it was not performed. The cases, if successful, would not have proved the correctness of the theory sought to be established by the operation, and were not accepted.

Dr. Sims decided that "normal ovariotomy" is a misnomer by Dr. Battey, and has named it "Battey's operation." The change seems to have been necessary, as the operation approved by him and others is made for diseased ovaries. In fact, an unhealthy condition of the ovaries is proved generally to exist where the operation has been found useful. Now, if the removal of diseased ovaries constitutes the procedure in question, as seems to be the case, it would look more like justice to Kentucky's favorite to call it McDowell's operation, as that distinguished surgeon was the first who performed the operation in America.

